# Nosocomial valve endocarditis due to corynebacterium striatum: a case report

**DOI:** 10.1186/1757-1626-1-388

**Published:** 2008-12-12

**Authors:** Jorge Marull, Pablo A Casares

**Affiliations:** 1 515 59th St. Apt 25C 25th FL, zip 10019, New York, NY, USA; 2 10 Amsterdam Ave. Apt 306, zip 10023, New York, NY, USA; 3 Department of Medicine, St Luke's Roosevelt Hospital Center, Columbia University College of Physicians and Surgeons, New York, NY, USA; 4 St. Luke's Roosevelt Hospital Center, Amsterdam Ave & W 113th St New York, NY 10025, USA

## Abstract

Staphylococcus aureus, Coagulase-negative staphylococci, and Enterococci sp. are the usual pathogens involved in nosocomial bacterial endocarditis. Corynebacterium species isolation in blood specimens is usually considered to be a contaminant. We present an interesting case of native mitral valve endocarditis in a 73 year old African American female that was diagnosed days after she was discharged from our institution. The infection was cleared with medical therapy alone.

## Introduction

Over the past twenty years, the epidemiological profile of infective endocarditis has been changing. [1] The number of cases of hospital-acquired endocarditis has been increasing. This entity carries a higher mortality rate compared with community-acquired cases. Indwelling catheters, intravenous lines, intravascular procedures and devices have increased the risk of bacteriemia. [2] The presentation is not always evident and especial importance should be given to the clinical history in a patient with fever and no evident source of infection. Positive blood cultures for Corynebacterium species, and Corynebacterium striatum in particular, should never be overlooked.

## Case report

This is a case of a 73-year-old African American female with a history of hypertension, chronic kidney disease, diastolic congestive heart failure and diabetes mellitus who consulted to the Emergency Service with three-day history of fevers, chills and fatigue. The patient was discharged home 15 days prior to this admission after a 9-day hospitalization where she received treatment for an exacerbation of her heart failure. Vital signs on this admission: Temperature of 102.4 F, Blood pressure of 170/68 mm Hg, Heart Rate of 100 beats per minute, Respiratory Rate of 20 breaths per min., and Oxygen Saturation of 98% on 3 liters nasal canula. Physical examination revealed a new 3/6 non-radiating systolic murmur at the apex. Auscultation of the lungs revealed clear breath sounds and inspection of lower extremities showed trace edema bilaterally. Basic metabolic panel and complete blood cell count was ordered (table 1). The patient was started on broad spectrum coverage with Vancomycin and Cefepime; due her previous admission, she was treated as she was having a nosocomial infection. Two sets of blood cultures drawn on admission grew CS in both the aerobic and anaerobic bottles sensitive to vancomycin (table 2). In order to distinguish CS from other Corynebacterium species, a metabolic profile obtained in the bioMerieux data base (version 2.0) was applied. Surveillance cultures were negative on day 6. A trans-thoracic echocardiogram showed asymmetric shaggy densities on the mitral valve, suggesting possible vegetations. The Doppler study revealed mild to moderate mitral regurgitation, and a normal ejection fraction alone with an abnormal diastolic compliance of the left ventricle. A similar study done on the previous admission did not reveal any foreign material or valvular damage. A subsequent trans-esophageal echocardiogram confirmed the presence of a 2.9 × 0.5 cm echo densities indicating mitral valve endocarditis with possible small eccentric perforation of the valve. The patient received a six-week course of intravenous Vancomycin, dosed according to through blood levels with a target of 15–20 ug/ml, and became afebrile. She recovered and was finally discharged home. Surgical repair of the mitral valve was offered, which she adamantly refused. A surveillance echocardiogram done after treatment unfortunately showed findings consistent with moderate pulmonary hypertension and a persistent mitral regurgitation.

**Table 1:** Basic metabolic panel and complete blood cell count

**Hematology**		**Chemistry**			
WBC	13.300	Sodium	138	ANA	negative
Hemoglobin	8.3	Potasium	4.2	Comp C3	normal
Hematocrit	25	Chloride	109	Comp C4	normal
Platelet	334	BUN	65	Hepatitis panel non-reactive	
		Creatinine	3.0		
		Glucose	183		

Ref: White blood count (WBC), Blood Urea Nitrogen (BUN).

**Table 2:** Antibiotic resistance profile

Ampicillin	> 4 R
Azithromycin	> 2 R
Cefepime	> 2 R
Cefotaxime	> 2 R
Erythromycin	>0.5 R
Meropenem	0.5 S
Vancomycin	0.5 S

Ref: S = SUSCEPTIBLE. I = INTERMEDIATE R = RESISTANT IR = INDUCED RESISTANT.

## Discussion

Two of the Duke major criteria were met, including the microbiology and echocardiographic findings, making a definitive diagnosis. [3] There is neither a recognized standard method nor specific MIC breakpoints or guidelines published by NICCS for antimicrobial testing of coryneform bacteria. National Committee for Clinical Laboratory Standards interpretive criteria currently valid for staphylococci, streptococci, or listeria monocytogenes was applied. Chester in 1901 and then Eberson in 1918 described for the first time the Striatum strain, and since then numerous corynebacterium species have been identified and reclassified, in different strains. [4]

In the 1980s and 1990s CS infections have been disregarded. It was only until recently that Corynebacterium diphtheria was considered to be the only pathogen of the coryneform species. Today, other species, such as C. jeikeium, C. urealiticum, C. amycolatum along with CS, are recognized as emerging hospital pathogens. The pathogenicity in non-diphtheriae corynebacteria species isn't well-known. Neither toxin nor other virulence factors explain the transition from colonization to infection. [5] Martin et al. isolated identical strains of CS from a leg ulcer and from the bloodstream in a patient with peripheral vascular disease, confirming the entry of the bacterium through the skin to the circulation. [6] The spectrum of diseases generated by CS is extensive, going from meningitis, keratitis, to arthritis, among others. [5]

The spread of CS from patient to patient and nosocomial outbreaks have been documented. Leonard et al., Iaria et al., and Brandenbrug described outbreaks caused by a single strain in intensive care unit settings. Leonard et al., described a selective pressure due to prior exposure to antibiotics favoring the overgrowth of CS in immune deficient patients. Brandenbrug et al recovered the strain in the air around infected patients and on the hands of the housestaff. [7-9]. Vancomycin is still the drug of choice for many authors since in vitro resistance has not been showed for this or any other Corynebacterium species. The spread of multidrug resistance strains of CS is concerning, although the mechanisms are not cleared. Ten years ago, CS was still sensitive to beta lactams, fluoroquinolones, carbapenems and linezolid. [10,11]

## Conclusion

In the hospital setting, positive blood cultures for Corynebacterium species, and CS in particular, should never be overlooked. Unlike other hospital-acquired pathogens such as staphylococcal or enterococcal species, that tend to infect patients with a predisposing condition such as a valvulopathy, CS endocarditis can be seen even in patients without prosthetic valves or structural heart disease. More research needs to be conducted to evidence what resistance mechanisms are involving this organism. Nowadays CS is being considered one of the emergent nosocomial agents implicated in endocarditis and serious infections.

## Consent

Written informed consent was obtained from the patient for publication of this case report and accompanying images. A copy of the written consent is available for review by the Editor-in-Chief of this journal.

## Competing interests

The authors declare that they have no competing interests.

## Authors' contributions

JM was a major contributor in writing the manuscript, analyzing the data, and collecting the information in the outpatient setting. PC wrote the abstract and made a mayor contribution in the discussion and conclusion. Reviewed the article and corrected the medical terminology used. Both authors read and approved the final manuscript.
